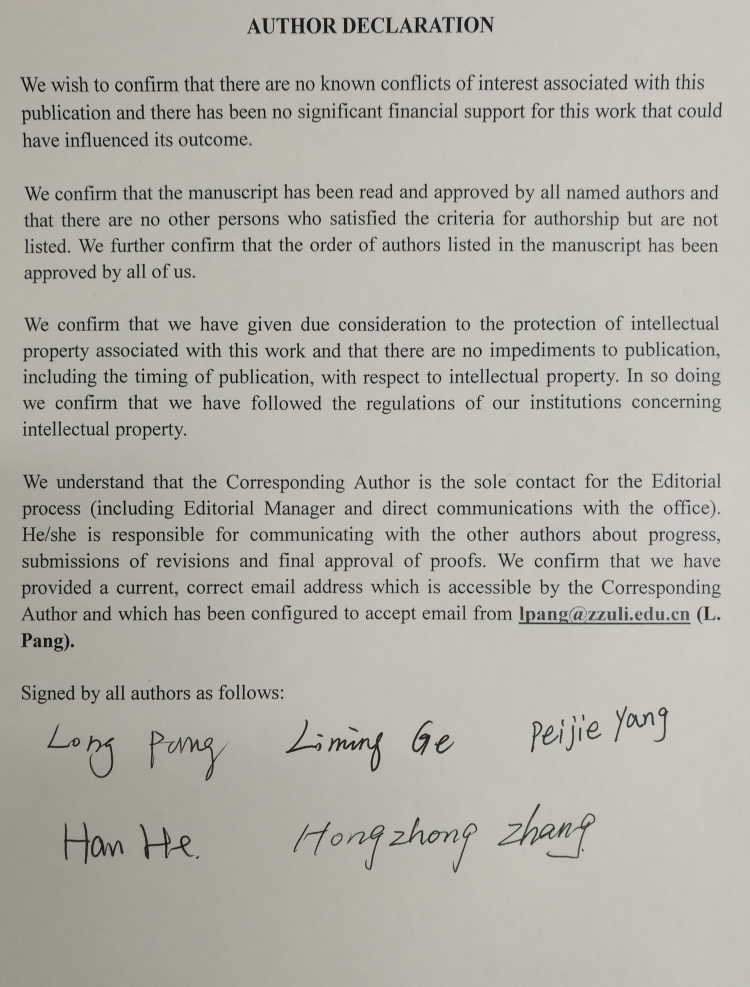# Degradation of organophosphate esters in sewage sludge: Effects of aerobic/anaerobic treatments and bacterial community compositions

**DOI:** 10.1016/j.dib.2018.02.039

**Published:** 2018-02-16

**Authors:** Long Pang, Liming Ge, Peijie Yang, Han He, Hongzhong Zhang

**Affiliations:** a Department of Material and Chemical Engineering, Zhengzhou University of Light Industry, Zhengzhou 450001, People's Republic of China; b Collaborative Innovation Center of Environmental Pollution Control and Ecological Restoration, Zhengzhou 450001, People's Republic of China

## Abstract

This dataset provides detail information on the analytical methods of organophosphate esters (OPEs) in sludge samples, including the sample preparation, ultra-high performance liquid chromatography-tandem mass spectrometric (UPLC-MS/MS) analysis, quality assurance and quality control (QA/QC). The concentration of target OPE compounds in collected samples of four individual treatment was provided, including aerobic composting combined with pig manure (T1), aerobic composting without pig manure (T2), anaerobic digestion combined with pig manure (T3), and anaerobic digestion without pig manure (T4). To investigate the variation of bacterial community compositions, principal components analysis (PCA) was provided based on the high-throughput sequencing. These data would be useful for clarifying the removal of OPEs under aerobic and anaerobic conditions. Besides, it also provides important information on the potential bacterial strains responsible for the biodegradation of OPEs in each treatment.

**Specifications Table**

**Table:** 

Subject area	Microbiology
More specific subject area	Composting, digestion, organic pollutants, biodegradation
Type of data	Tables and figures
How data was acquired	The parameters during the composting process were obtained from Compsoft 3.0. The concentration of target OPEs was measured by using an UPLC-MS/MS (Ultimate 3000, Thermo Scientific, USA) system equipped with a triple quadrupole mass spectrometer (TSQ Endura, Thermo Scientific, USA). Principal components analysis was conducted in R with the package “gplots”.
Data format	Raw data collection and analysis.
Experimental factors	The sewage sludge samples were pretreated with a Dionex ASE 350 system coupled with an Oasis HLB cartridge. Each sample was spiked with 10 μL of TnBP-d27 at 5 mg L^−1^ as surrogate before extraction.
Experimental features	Four individual experiments were carried out for evaluating the degradation of OPEs and the variation of bacterial community compositions.
Data source location	Zhengzhou, People's Republic of China
Data accessibility	Data are presented in this article

**Value of the data**

• The dataset provides the detail information on the analytical method, including sample preparation, UPLC-MS/MS analysis, and QA/AC.
• The concentration of OPEs in each sampling would be useful to understand the removal rate and make a comparison among different treatments.
• To understand the variation of bacterial community compositions, abundance, and diversity in sewage sludge with different conditions.
• To identify the potential bacteria responsible for the degradation of OPEs in sewage sludge with different conditions.

## 1 Data

**Fig. 1:**
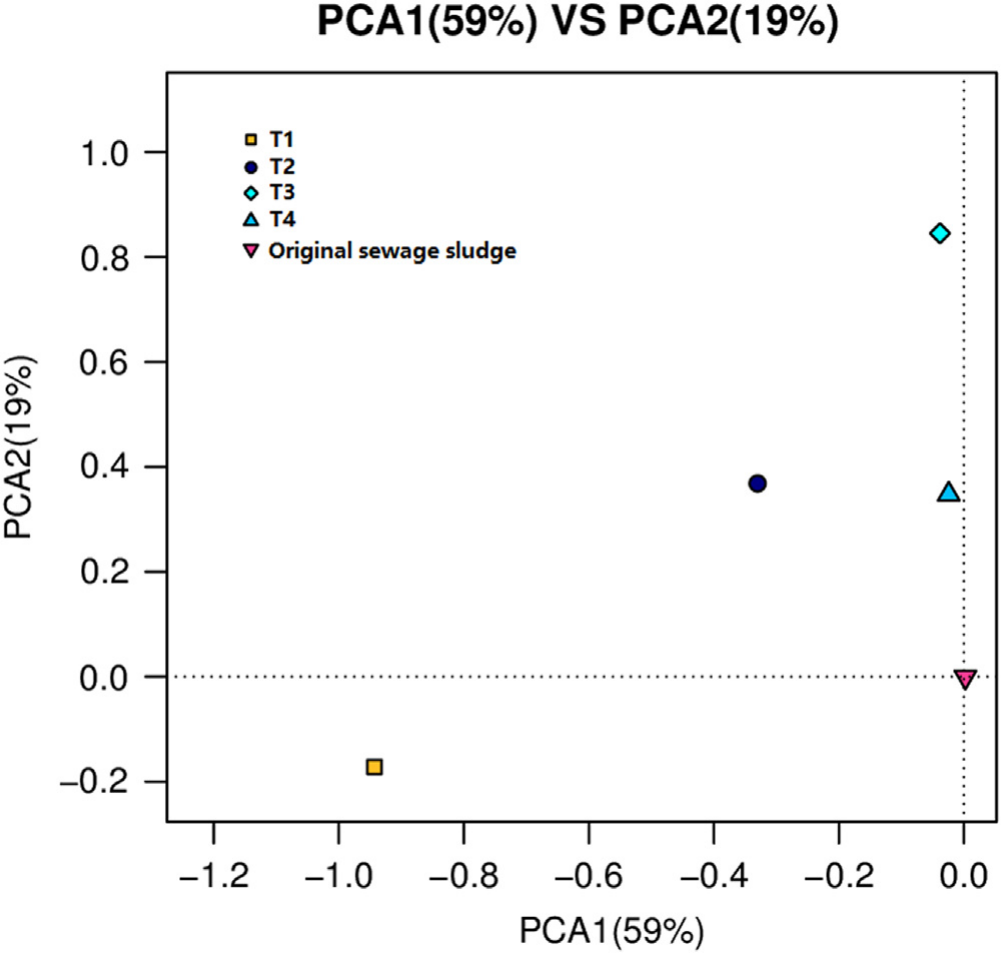
The loading plots of principal components analysis (PCA) based on the bacterial community compositions detected in the sludge samples.

Organophosphate esters (OPEs) are widely used as flame retardants and plasticizers in recent years [1]. Because of the potential risks for human health, OPEs are regarded as a class of emerging pollutants [2], [3]. High concentration levels of OPEs were found in the dewatered sewage sludge because of the adsorption on the activated sludge during the wastewater treatment process [4]. Composting is an effective way to realize the sludge recycling and harmless disposal [5], [6]. The matrix in the composts was complex and the spiked recoveries were usually low. Accelerated solvent extraction combined with solid phase extraction method was used for the determination of OPEs in this study. Detail information was provided in our previous work [7]. The concentration of OPEs in collected samples during the whole process was listed in Table 1, Table 2, Table 3, Table 4. The principal components analysis was shown in Fig. 1.

**Table 1:** Observed removal of target OPEs in aerobic composting combined pig manure.

Sample	Sampling time (d)	TnBP (ng g^−1^, dw)	TCEP (ng g^−1^, dw)	TPhP (ng g^−1^, dw)	TCPP (ng g^−1^, dw)	TBEP (ng g^−1^, dw)	TCrP (ng g^−1^, dw)	∑OPEs (ng g^−1^, dw)
#1	0.25	23.71±0.02	34.68±0.42	21.18±0.11	34.05±0.54	9.03±0.04	3.24±0.03	125.90
#2	0.50	28.82±0.08	20.62±0.48	20.73±0.02	40.42±0.45	9.74±0.08	2.77±0.04	123.11
#3	0.75	29.56±0.79	21.34±0.49	20.85±0.24	53.40±0.71	8.73±0.05	2.74±0.01	136.61
#4	1.00	25.01±0.11	23.93±0.77	21.21±0.01	37.74±0.17	9.36±0.06	2.74±0.02	119.98
#5	1.25	24.38±0.10	18.52±0.15	20.34±0.26	31.89±0.13	9.68±0.08	2.78±0.03	107.58
#6	1.75	35.56±0.11	19.29±0.43	22.09±0.09	29.97±0.76	8.20±0.02	2.83±0.02	117.94
#7	2.25	27.64±0.18	17.26±0.32	19.58±0.09	30.63±0.41	8.22±0.09	2.73±0.02	106.07
#8	3.25	30.70±0.16	18.00±0.35	20.27±0.27	32.25±0.53	8.32±0.04	2.71±0.01	112.26
#9	4.25	31.34±0.16	18.34±0.08	19.53±0.17	21.65±0.52	7.73±0.08	2.83±0.02	101.41
#10	5.25	20.46±0.09	16.04±0.14	19.18±0.07	29.96±0.27	6.82±0.09	2.61±0.01	95.08
#11	6.25	25.64±0.20	17.50±0.46	20.08±0.10	32.31±0.87	7.56±0.06	2.84±0.03	105.93
#12	7.25	24.43±0.18	19.30±1.18	19.38±0.01	37.24±0.20	7.51±0.03	2.90±0.05	110.77
#13	8.25	28.29±0.33	19.92±0.46	20.24±0.11	32.39±0.30	7.77±0.04	2.89±0.03	111.50
#14	9.25	20.10±0.17	16.31±0.14	19.92±0.02	24.64±0.17	7.06±0.02	2.59±0.02	90.60
#15	10.25	30.03±0.07	15.91±0.29	19.19±0.21	25.09±1.39	6.59±0.30	2.73±0.03	99.53
#16	11.25	32.46±0.34	20.48±0.50	19.65±0.10	35.51±0.31	8.15±0.02	2.64±0.02	118.89
#17	12.25	36.38±0.47	16.95±0.22	18.61±0.12	27.44±0.40	8.12±0.03	2.59±0.03	110.09
#18	13.25	33.42±0.27	17.52±0.20	18.63±0.09	28.08±0.14	7.66±0.07	2.59±0.02	107.90
#19	14.25	31.60±0.15	18.29±0.92	18.71±0.06	23.08±0.26	7.58±0.03	2.64±0.02	101.90
Δ*m* (%)		−33.28%	47.26%	11.66%	32.22%	16.06%	18.52%	19.06%

**Table 2:** Observed removal of target OPEs in aerobic composting.

Sample	Sampling time (d)	TnBP (ng g^−1^, dw)	TCEP (ng g^−1^, dw)	TPhP (ng g^−1^, dw)	TCPP (ng g^−1^, dw)	TBEP (ng g^−1^, dw)	TCrP (ng g^−1^, dw)	∑OPEs (ng g^−1^, dw)
#1	0.25	32.37±0.10	20.66±0.13	20.92±0.04	15.88±0.26	7.68±0.03	3.10±0.03	100.61
#2	0.50	28.23±0.11	17.69±0.52	23.51±0.04	17.38±0.15	7.74±0.06	2.55±0.01	97.11
#3	0.75	31.64±0.24	17.38±0.19	22.36±0.71	16.18±0.18	7.73±0.03	2.83±0.01	98.06
#4	1.00	28.23±0.20	17.41±0.13	20.38±0.02	19.52±0.36	7.93±0.02	2.61±0.03	96.08
#5	1.25	35.64±0.38	19.54±0.19	19.96±0.09	22.01±0.46	8.50±0.10	2.65±0.01	108.30
#6	1.75	29.40±0.09	19.15±0.47	23.47±0.17	25.96±0.42	8.27±0.07	2.80±0.04	109.06
#7	2.25	26.47±0.32	17.15±0.41	21.37±0.18	26.85±0.04	8.11±0.07	2.76±0.02	102.72
#8	3.25	21.84±0.16	16.77±0.78	20.92±0.18	19.32±0.21	7.87±0.04	2.55±0.01	89.26
#9	4.25	30.83±0.12	24.33±0.89	20.85±0.03	21.17±0.47	8.10±0.05	2.95±0.01	108.24
#10	5.25	31.16±0.08	19.50±0.35	19.99±0.04	22.67±0.14	7.84±0.10	2.70±0.00	103.86
#11	6.25	31.81±0.20	17.21±0.78	58.92±0.34	27.21±0.37	8.19±0.03	2.64±0.02	145.96
#12	7.25	27.99±0.32	18.51±0.30	19.46±0.08	28.00±0.51	7.74±0.06	2.71±0.01	104.41
#13	8.25	19.73±0.34	20.04±0.38	19.82±0.02	33.16±0.39	6.88±0.03	2.84±0.01	102.49
#14	9.25	30.17±0.34	18.67±0.17	18.63±0.04	23.06±0.34	8.10±0.03	2.70±0.03	101.34
#15	10.25	30.04±0.20	23.75±0.33	19.83±0.08	27.65±0.18	8.50±0.13	2.54±0.03	112.32
#16	11.25	39.36±0.05	18.02±0.13	19.01±0.11	26.95±0.31	8.81±0.01	2.70±0.03	114.85
#17	12.25	27.23±0.20	17.17±0.50	19.36±0.11	26.24±0.56	12.83±0.07	2.80±0.01	105.63
#18	13.25	26.40±0.18	24.94±0.48	19.40±0.15	21.74±0.58	7.05±0.02	2.78±0.02	102.30
#19	14.25	16.47±0.09	29.06±0.53	19.44±0.04	23.87±0.44	6.06±0.04	2.61±0.01	97.51
Δ*m* (%)		49.12%	−40.66%	7.07%	−50.31%	21.09%	15.81%	3.08%

**Table 3:** Observed removal of target OPEs in anaerobic digestion combined with pig manure.

Sample	Sampling time (d)	TnBP (ng g^−1^, dw)	TCEP (ng g^−1^, dw)	TPhP (ng g^−1^, dw)	TCPP (ng g^−1^, dw)	TBEP (ng g^−1^, dw)	TCrP (ng g^−1^, dw)	∑OPEs (ng g^−1^, dw)
#1	0.25	24.84±0.40	33.96±0.57	20.93±0.12	37.79±0.30	8.34±0.12	2.84±0.03	128.70
#2	0.50	24.33±0.37	23.40±1.00	19.56±0.10	25.53±0.39	7.99±0.03	2.64±0.04	108.45
#3	0.75	25.70±0.14	22.26±0.26	19.02±0.07	22.44±0.53	7.86±0.05	2.62±0.04	99.91
#4	1.00	17.89±0.29	24.14±0.86	19.78±0.13	26.73±0.44	7.78±0.02	2.82±0.08	99.14
#5	1.25	18.47±0.09	23.88±0.54	19.28±0.05	24.68±0.26	7.98±0.12	2.70±0.04	97.00
#6	1.75	18.53±0.09	19.33±0.51	19.08±0.13	23.76±0.18	7.82±0.06	2.52±0.02	91.03
#7	2.25	21.84±0.12	20.24±0.75	18.51±0.52	25.14±0.59	7.87±0.04	2.60±0.05	96.20
#8	2.75	20.29±0.08	18.79±0.92	19.35±0.08	21.94±0.52	7.95±0.06	2.56±0.04	90.89
#9	3.75	22.82±0.04	19.22±0.18	19.15±0.06	29.92±1.06	7.13±0.04	2.53±0.03	100.37
#10	4.75	20.56±0.18	22.53±0.71	18.57±0.04	25.77±0.26	7.95±0.07	2.54±0.04	97.94
#11	5.75	25.32±0.11	16.51±0.18	19.07±0.03	24.34±0.12	8.91±0.03	2.68±0.02	96.83
#12	6.75	19.71±0.16	20.57±0.62	18.96±0.07	25.75±0.21	8.20±0.04	2.63±0.03	95.82
#13	7.75	24.50±0.16	24.51±0.20	19.43±0.07	26.38±0.39	9.13±0.06	2.75±0.02	106.71
#14	8.75	21.47±0.32	27.92±0.31	18.90±0.05	20.69±0.14	6.84±0.05	2.59±0.05	98.40
#15	9.75	23.18±0.03	23.78±0.27	18.66±0.08	20.61±0.33	6.30±0.03	2.62±0.04	95.16
#16	10.75	14.54±0.32	34.10±0.90	17.95±0.09	16.92±0.19	5.54±0.02	2.52±0.04	91.57
Δ*m* (%)		41.47%	−0.41%	14.24%	55.23%	33.57%	11.27%	28.85%

**Table 4:** Observed removal of target OPEs in anaerobic digestion.

Sample	Sampling time (d)	TnBP (ng g^−1^, dw)	TCEP (ng g^−1^, dw)	TPhP (ng g^−1^, dw)	TCPP (ng g^−1^, dw)	TBEP (ng g^−1^, dw)	TCrP (ng g^−1^, dw)	∑OPEs (ng g^−1^, dw)
#1	0.25	20.92±0.05	18.24±0.43	20.14±0.09	22.87±0.07	7.88±0.04	2.66±0.00	92.71
#2	0.50	25.31±0.09	17.21±0.33	20.52±0.13	25.41±0.63	8.97±0.11	3.09±0.03	100.51
#3	0.75	18.32±0.16	19.15±0.35	18.68±0.02	22.61±0.31	7.35±0.02	2.51±0.02	88.62
#4	1.00	16.56±0.07	18.04±0.27	19.13±0.04	19.70±0.31	7.60±0.05	2.83±0.01	83.86
#5	1.25	14.50±0.24	16.95±0.08	20.08±0.02	53.27±0.64	7.33±0.03	2.62±0.03	114.75
#6	1.75	12.02±0.04	16.97±0.55	19.20±0.08	18.48±0.43	6.82±0.05	2.50±0.00	75.99
#7	2.25	14.05±0.06	16.90±0.17	18.95±0.06	18.13±0.29	7.55±0.12	2.48±0.01	78.07
#8	2.75	20.20±0.21	19.15±0.70	21.35±0.08	33.94±0.40	9.08±0.05	2.63±0.02	106.34
#9	3.75	27.13±0.31	19.87±1.07	20.55±0.11	30.44±1.90	9.79±0.07	2.91±0.03	110.60
#10	4.75	30.59±0.10	21.82±0.57	22.08±0.09	35.40±1.11	12.34±0.24	3.37±0.03	125.60
#11	5.75	29.02±0.20	22.71±0.87	21.07±0.14	32.78±0.95	9.45±0.04	3.33±0.06	118.38
#12	6.75	28.63±0.02	26.41±0.26	20.41±0.09	35.88±0.55	9.84±0.04	2.95±0.03	124.12
#13	7.75	28.79±0.02	25.72±0.41	27.44±0.09	20.42±0.47	8.53±0.12	3.11±0.09	114.00
#14	8.75	23.31±0.25	31.14±1.09	21.0±0.04	57.11±0.53	10.43±0.05	2.92±0.01	146.01
#15	9.75	25.19±0.25	38.21±1.94	20.66±0.20	17.60±0.30	6.25±0.05	3.46±0.01	111.37
#16	10.75	26.24±0.13	31.89±0.51	19.91±0.12	15.47±0.40	6.41±0.06	3.00±0.036	102.92
Δ*m* (%)		−25.43%	−74.84%	1.14%	32.36%	18.65%	−12.78%	−11.01%

## 2 Experimental design, materials, and methods

### 2.1 Sample preparation

Briefly, the extraction procedure was performed on a Dionex ASE 350 system (Sunnyvale, CA, USA). Small amount of diatomaceous earth and 0.5 g of sample were loaded into a 33 mL capacity stainless steel cell. Each sample was spiked with 10 μL of TnBP-d_27_ at 5 mg L^−1^ as surrogate before extraction. Additional diatomaceous earth was added to fill the remaining free space of the cell. Two pieces of cellulose filter were placed on the bottom and top of the extraction cell, respectively. After ASE procedure, the extract was evaporated to almost dryness by using a rotary evaporator. The extract was re-dissolved in 6 mL of ACN and diluted to 200 mL with ultrapure water. The solution was filtered by GF/C membrane (glass fiber, 1.2 μm, 45 mm, Whatman, UK) and then subjected to an Oasis HLB cartridge (200 mg, 6 mL). The analytes were eluted by 8 mL of acetonitrile (ACN) and then concentrated to nearly dryness. The residue was redissolved in 1.5 mL ACN/water (40/60, v/v) and 5 μL of the solution was injected into UPLC-MS/MS for analysis.

### 2.2 UPLC-MS/MS analysis

A UPLC system (Ultimate 3000, Thermo Scientific, USA) equipped with a triple quadruple mass spectrometer (TSQ Endra, Thermo Scientific, USA) was used for the determination and identification of OPEs. The separation of analytes was performed on a Hypersil GOLD C18 (2.1 mm×50 mm, 1.9 μm). A binary mobile phase of an aqueous solution of 0.1% formic acid (A) and ACN containing 0.1% formic acid (B) at a flow rate of 0.3 mL min^−1^ was applied. The gradient was set as follows: 0 min (40% B), 0.5 min (40% B), 3 min (50% B), 4.5 min (55%, B), 8.5 min (70% B), 9 min (100% B), 13.8 min (100% B), 13.9 min (40% B), 17 min (40% B).

For MS/MS analysis, the electrospray ionization (ESI) was run in the positive ion mode. The optimal conditions were set as follows: peak width resolution 0.7 *m*/*z*, spray voltage 4500 V, sheath gas pressure 35 units, auxiliary gas pressure of 20 units, and capillary temperature 300 °C.

### 2.3 QA/QC

Field blanks (*n* = 3), procedural blanks (*n* = 3), spiked blanks (*n* = 3), spiked matrix (*n* = 3), and replicate samples (*n* = 7) were analyzed with extraction to control contamination. In each spiked sample, 50- and 100-ng mixture of OPEs were added. All samples were spiked with TnBP-d_27_ as surrogate. TCEP was not found in the blank; TnBP, TPhP, TCPP, and TBEP were detected at 2.95, 9.38, 3.90, and 2.00 g L^−1^ in the blank. The recoveries of standards in spiked samples were within 56–113% at two different spiked concentration levels. The matrix effect was evaluated by addition of standards into the pre-extracted samples were in the range of 83–121% at two different spiked concentration levels. Each batch of ten samples included one procedural blank to check potential contamination. All glassware was solvent rinsed and heated overnight at 400 °C before usage.

## Figures and Tables

**Fig. S1:**